# Research on fast marking method for indicator diagram of pumping well based on K-means clustering

**DOI:** 10.1016/j.heliyon.2023.e20468

**Published:** 2023-09-27

**Authors:** Xiang Wang, Zhiwei Shao, Yancen Shen, Yanfeng He

**Affiliations:** School of Petroleum and Natural Gas Engineering, Changzhou University, Changzhou, 213164, China

**Keywords:** Pumping well, Indicator diagram, Fault diagnosis, K-means clustering

## Abstract

Indicator diagram is the key basis for fault diagnosis of pumping wells in oil exploitation. With the rapid development of machine learning, the fault diagnosis of indicator diagram based on deep learning has garnered increasing attention. This kind of methods train neural network models with marked samples, and then inputs images into the trained models and outputs their categories. At present, the preparation of indicator diagram sample set relies on experts' analysis of indicator diagram images one by one. However, it involves extensive manual work and manual marking is prone to errors, so the marked samples are often insufficient in quantity. In order to quickly mark a large number of indicator diagram samples, the oil well data was plotted into standardized indicator diagram, and then three feature extraction methods for indicator diagrams were proposed: feature extraction based on original vector, feature extraction based on three-dimensional pixel tensor, feature extraction based on convolutional neural network. These methods convert the indicator diagram into corresponding feature vectors, which are then clustered using the K-means clustering algorithm, enabling the corresponding indicator diagrams to be classified into different categories based on the clustering results. Using 20,000 randomly selected pieces of data from 100 pumping wells, this study clusters the sample set using the three proposed methods. The results indicated that the time consumption were 0.2, 8.3, and 0.7 h, with accuracy rates of 98%, 92%, and 95%, respectively. For indicator diagrams, the clustering method based on the original vector has outstanding performance in terms of efficiency and accuracy. This provides an automatic tool for the preparation of the pumping well fault diagnosis dataset, and its efficiency can be increased by tens of times compared with manual marking.

## 1 Introduction

The pumping unit serves as the main equipment for oil exploitation [1], and the fault diagnosis of pumping unit is one of the crucial issues in the field of oil exploration and development. Indicator diagram is composed of a series of displacement and load sample points when the pumping unit is in operation [2], which serves as the key basis for fault diagnosis of pumping unit. Researchers have extensively investigated the problem of pumping well fault diagnosis using two main approaches. The first is the method based on theoretical analysis [3], which involves establishing the operating principles and corresponding mathematical models of the sucker rod pumping system, followed by the simulation calculation of various pumping well fault types. However, given the complexity of the rod pumping system and the interactions among various fluids in the wellbore, this method has limited applications. The second is based on machine learning [4], which involves preparing a series of sample sets based on different shapes of indicator diagrams and corresponding fault types. Subsequently, the appropriate machine learning model is selected to learn the sample set, facilitating the accurate identification of the fault. This approach avoids the need for complex physical mechanism description and has emerged as the mainstream method for pumping well fault diagnosis.

With the advancement of machine learning methods, deep learning has emerged as a powerful tool and its application has been expanding rapidly [5]. The methods based on deep learning have demonstrated significant potential and prospects in the field of fault diagnosis. [6]. Jia et al. employed a Deep auto-encoder for intelligence diagnosis of rotating machinery [6], Gan and Wang utilized deep belief network for fault pattern recognition of rolling element bearings [7], Zhao et al. adopted recurrent neural network for machine health monitoring [8], Zhang et al. applied convolutional neural network for bearing fault diagnosis [9], applied convolutional neural network for bearing fault diagnosis. Notably, convolutional neural network has emerged as the leading architecture and has exhibited the best performance in numerous tests [10]. In recent years, deep learning has also been extensively applied to fault diagnosis of pumping units [[11], [12], [13], [14]].

The fault diagnosis method for pumping wells based on deep learning heavily relies on the quantity and quality of the sample set. In current research, the number of samples usually ranges from hundreds to tens of thousands, with fault types mostly ranging from several to dozens. In order to verify the proposed method, Feng et al. collected 700 samples of five typical faults (discharge valve or pipe blockage, suction valve or pipe blockage, suction valve leakage, discharge valve leakage and discharge and suction valve leakage.) and extracted the required features [11]. Cheng et al. considered eight different fault diagnosis types, which include normal operation condition, downstroke pump bumping, upstroke pump bumping, combination of leaking standing and traveling valves, gas interference, insufficient liquid supply, sand production and abnormal dynamometer card. For each type, they selected 1000 samples for identification and classification [12]. Du et al. compared different models on 18,500 manually marked fault samples of pumping units in ten categories [15]. Lv et al. collected 7887 indicator diagrams under different working conditions from Shengli Oilfield in China to prove the effectiveness and performance of the proposed fault diagnosis method, and each sample was manually marked [13]. Zhang et al. used displacement load data from an oil plant to create indicator diagrams and manually marked 7500 data sets of five fault types [16]. Wei et al. collected 2000 indicator diagrams of 12 fault types in Y Oilfield and manually marked each diagram before diagnosis Wei et al. collected 2000 indicator diagrams of 12 fault types in Y Oilfield and manually marked each diagram before diagnosis [17]. A summary of the above studies is shown in Table 1.

**Table 1:** A summary of related works.

Publication	Diagnosis algorithm	Dataset		
		Marking method	Types of faults covered	Number of samples
[11]	SVM	manual	5	700
[12]	SVM	manual	8	1000
[15]	Attention CNN	manual	10	18500
[13]	SVM	manual	13	7887
[16]	Capsule Neural Network	manual	5	7500
[17]	CNN + PSO	manual	12	2000
[14]	CNN	manual	8	13804

With the construction of oilfield intelligence, automatic data acquisition has become a reality. Massive data has been accumulated to form big data. For instance, Shengli Oilfield in China generates an average of 48 indicator diagrams per well per day, resulting in hundreds of millions of indicator diagrams each year [18]. These indicator diagrams cover almost all types of faults that may occur in the operation of pumping wells. However, manually marking such an enormous amount of data is unfeasible, which leads to the inability of massive information to play its value. Consequently, the sample size has emerged as a critical challenge that hinders fault diagnosis. The conventional method of manual marking on a one-by-one basis is inadequate for batch marking of massive sample data.

The clustering analysis method in unsupervised learning is being increasingly applied to various fields [19,[20], [21], [22], [23], [24], [25]], which helps us make full use of a large number of unmarked samples and saves a lot of manual work and time. There are also many applications that combine clustering analysis with machine learning algorithms. Hao utilized clustering to analyze physique data of college students, and subsequently trained a BP neural network [26]). Li et al. utilized K-means clustering to classify grape leaves, and subsequently used a random forest algorithm to detect and grade grape downy mildew [27]. Ida et al. applied K-means clustering to classify and study the waveform of volcanic earthquakes, and used their findings to observe magma rising events in Sakurajima [28].

At present, a large number of unmarked samples are not fully utilized in the fault diagnosis of pumping wells, and sample labeling requires a lot of manual labor. In order to realize the automatic and fast clustering of pumping well indicator diagrams, we introduced the K-means clustering method, and compared the clustering performance of three different dynamometer image feature extraction methods, including feature extraction based on original vector, feature extraction based on three-dimensional pixel tensor, and feature extraction based on convolutional neural network. This study selects 20,000 samples randomly and conducts comparative experiments to analyze the efficiency and accuracy of the aforementioned methods.

The remainder of this paper is organized as follows. Section 2 introduces the drawing process of standardized indicator diagram. Section 3 describes the feature extraction methods of indicator diagram. Section 4 introduces the clustering for indicator diagrams based on K-means algorithm. Section 5 presents the experiments results and analysis. Finally, Section 6 concludes the paper.

## 2 Standardized indicator diagram drawing

**Fig. 1:**
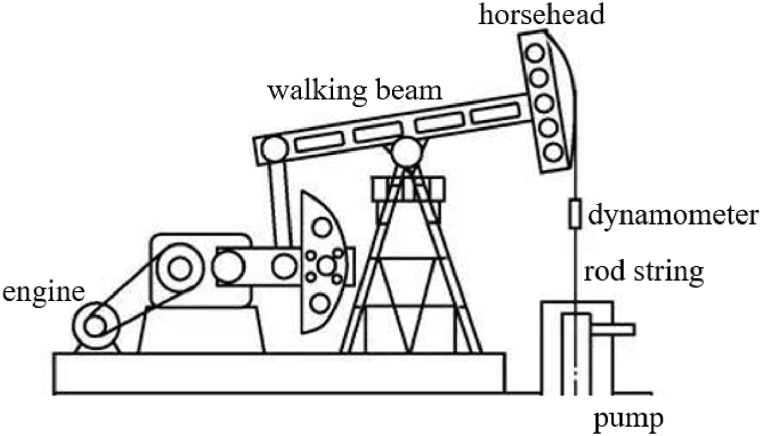
A typical pumping well system.

A typical pumping well system is shown in Fig. 1. The engine converts the rotary motion into the up and down reciprocating motion of the horsehead through the walking beam. The rod string is connected between the horsehead and the pump at the bottom of the well. A dynamometer is installed at the top of rod string. It can record the displacement and load in the movement of horsehead.

Usually, during a reciprocating movement up and down the horsehead, the dynamometer records 200 data points for displacement and load respectively, forming a displacement vector (**W** = [w_1_, w_2_, …, w_200_]) and a load vector (**Z** = [z_1_, z_2_, …, z_200_]). Through the informationization construction of oilfields, nearly all pumping wells are equipped with dynamometers, allowing for continuous collection and transmission of displacement and load data to the oilfield data center [29]. Table 2 presents a portion of the displacement and load vectors of one of the wells in an oilfield on July 10, 2021. Taking into account information transmission and storage capabilities, this oilfield collects displacement and load vectors every half hour per well.

**Table 2:** A portion of displacement and load data.

Acquisition time	Displacement/(m)	Load/(kN)
2022-07-09 06:14:48	0.0,0.006,0.003,0.009,0.025,0.038,0.054,0.063,0.092,0.111,0.134,0.172,0.194,0.21,0.258,0.281,0.325,0.364,0.383,0.437,0.475,0.504,0.562,0.6,0.654,0.709,0.769,0.808,0.859,0.923,0.974,1.041,1.092,1.149,1.213,1.277,1.331,1.402,1.453,1.517,1.577,1.648,1.705,1.769,1.833,1.887,1.97,2.015,2.079,2.146,2.2,2.267,2.318,2.373,2.44,2.504,2.558,2.619,2.679,2.721,2.778,2.842,2.871,2.941,2.986,3.037,3.098,3.142,3.181,3.229,3.334,3.372,3.411,3.452,3.487,3.538,3.577,3.631,3.657,3.689,3.724,3.752,3.781,3.823,3.845,3.871,3.896,3.925,3.947,3.986,3.992,4.017,4.049,4.059,4.059,4.078,4.088,4.113,4.12,4.126,4.136,4.139,4.155,4.145,4.148,4.148,4.152,4.155,4.145,4.136,4.129,4.12,4.104,4.091,4.069,4.037,4.027,4.005,3.976,3.944,3.912,3.88,3.845,3.813,3.768,3.759,3.701,3.657,3.612,3.561,3.516,3.462,3.401,3.353,3.312,3.241,3.181,3.114,3.053,2.995,2.938,2.887,2.81,2.737,2.682,2.612,2.536,2.472,2.411,2.341,2.286,2.207,2.13,2.076,2.009,1.938,1.871,1.801,1.727,1.676,1.6,1.529,1.475,1.402,1.338,1.268,1.226,1.159,1.098,1.025,0.977,0.916,0.862,0.808,0.753,0.705,0.645,0.587,0.552,0.504,0.45,0.402,0.37,0.345,0.284,0.252,0.223,0.194,0.178,0.147,0.115,0.086,0.063,0.057,0.057,0.057,0.032,0.016,0.012,0.012	35.99,36.49,37.17,37.93,38.67,39.58,40.58,41.81,43.14,44.61,46.09,47.65,49.38,51.15,53.09,55.0,57.12,59.36,61.71,64.16,66.24,67.57,68.04,68.04,68.07,68.19,68.16,68.07,68.1,68.16,68.25,68.3,68.42,68.57,68.54,68.51,68.46,68.54,68.6,68.66,68.6,68.6,68.57,68.54,68.6,68.69,68.87,68.87,68.93,68.93,68.99,68.99,68.99,69.04,69.04,69.01,69.01,69.01,69.04,68.99,68.99,68.87,69.04,69.31,69.48,69.48,69.34,69.43,69.31,69.31,69.16,69.19,69.13,69.13,69.07,68.99,68.84,68.78,68.81,68.87,68.81,68.63,68.54,68.46,68.43,68.34,68.28,68.22,68.16,68.13,68.07,68.01,67.93,67.9,67.84,67.75,67.6,67.48,67.31,67.16,66.98,66.8,66.57,66.28,65.98,65.54,65.1,64.54,64.04,63.45,62.92,62.45,61.95,61.42,60.74,60.12,59.42,58.74,57.92,57.06,56.09,55.0,53.77,52.41,50.91,49.23,47.35,45.41,43.35,41.05,38.61,36.13,34.63,33.93,33.96,33.96,33.96,34.02,34.05,34.05,34.02,33.96,33.9,33.87,33.93,33.93,33.87,33.69,33.64,33.49,33.4,33.31,33.37,33.34,33.37,33.31,33.43,33.4,33.4,33.4,33.43,33.49,33.49,33.55,33.61,33.64,33.58,33.52,33.4,33.43,33.43,33.52,33.49,33.52,33.55,33.58,33.61,33.66,33.69,33.69,33.61,33.69,33.72,33.81,33.78,33.78,33.78,33.81,33.87,33.96,34.05,34.14,34.25,34.34,34.4,34.52,34.72,35.02,35.34,35.61
2022-07-09 06:44:48	0.0,0.006,0.003,0.009,0.025,0.038,0.054,0.063,0.092,0.111,0.134,0.172,0.194,0.21,0.258,0.281,0.325,0.364,0.383,0.437,0.475,0.504,0.562,0.6,0.654,0.709,0.769,0.808,0.859,0.923,0.974,1.041,1.092,1.149,1.213,1.277,1.331,1.402,1.453,1.517,1.577,1.648,1.705,1.769,1.833,1.887,1.97,2.015,2.079,2.146,2.2,2.267,2.318,2.373,2.44,2.504,2.558,2.619,2.679,2.721,2.778,2.842,2.871,2.941,2.986,3.037,3.098,3.142,3.181,3.229,3.334,3.372,3.411,3.452,3.487,3.538,3.577,3.631,3.657,3.689,3.724,3.752,3.781,3.823,3.845,3.871,3.896,3.925,3.947,3.986,3.992,4.017,4.049,4.059,4.059,4.078,4.088,4.113,4.12,4.126,4.136,4.139,4.155,4.145,4.148,4.148,4.152,4.155,4.145,4.136,4.129,4.12,4.104,4.091,4.069,4.037,4.027,4.005,3.976,3.944,3.912,3.88,3.845,3.813,3.768,3.759,3.701,3.657,3.612,3.561,3.516,3.462,3.401,3.353,3.312,3.241,3.181,3.114,3.053,2.995,2.938,2.887,2.81,2.737,2.682,2.612,2.536,2.472,2.411,2.341,2.286,2.207,2.13,2.076,2.009,1.938,1.871,1.801,1.727,1.676,1.6,1.529,1.475,1.402,1.338,1.268,1.226,1.159,1.098,1.025,0.977,0.916,0.862,0.808,0.753,0.705,0.645,0.587,0.552,0.504,0.45,0.402,0.37,0.345,0.284,0.252,0.223,0.194,0.178,0.147,0.115,0.086,0.063,0.057,0.057,0.057,0.032,0.016,0.012,0.012	36.1,36.52,37.16,37.78,38.52,39.32,40.29,41.49,42.76,44.23,45.68,47.32,49.03,50.82,52.65,54.68,56.74,58.98,61.21,63.66,65.86,67.34,67.96,68.01,68.01,68.13,68.28,68.48,68.57,68.6,68.57,68.6,68.66,68.72,68.89,69.01,69.16,69.07,69.07,69.04,69.04,69.04,69.04,69.04,69.04,69.07,69.22,69.22,69.22,69.1,69.16,69.22,69.31,69.34,69.31,69.34,69.37,69.43,69.43,69.4,69.34,69.31,69.25,69.31,69.22,69.28,69.22,69.28,69.25,69.19,69.19,69.16,69.19,69.16,69.1,69.07,69.05,69.02,69.02,68.99,68.99,69.01,69.01,69.01,68.9,68.78,68.63,68.42,68.4,68.34,68.31,68.13,67.98,67.93,67.9,67.81,67.72,67.48,67.39,67.19,67.1,66.95,66.72,66.4,65.98,65.63,65.25,64.75,64.19,63.54,63.01,62.51,62.07,61.48,60.89,60.27,59.68,59.04,58.21,57.36,56.39,55.39,54.18,52.74,51.15,49.38,47.61,45.7,43.67,41.46,39.08,36.72,34.99,34.22,34.19,34.31,34.25,34.19,34.05,34.11,34.08,34.2,34.14,34.11,33.99,33.93,33.9,33.87,33.81,33.78,33.72,33.66,33.61,33.64,33.64,33.67,33.61,33.61,33.55,33.58,33.61,33.58,33.58,33.58,33.67,33.67,33.67,33.58,33.58,33.61,33.78,33.84,33.78,33.75,33.75,33.9,33.9,33.9,33.81,33.81,33.81,33.9,33.93,33.96,34.05,34.05,34.05,33.99,34.08,34.22,34.19,34.34,34.43,34.64,34.64,34.72,34.93,35.19,35.52,35.78
…	…	…

To facilitate manual analysis, displacement and load vectors are often plotted as indicator diagrams. Indicator diagram take displacement vector as x-axis and load vector as y-axis, forming a closed-loop curve. It shows the load changes of the sucker rod pump in a reciprocating cycle, which can reflect the working condition of the pumping well. In this study, all indicator diagrams are drawn with reference to the standards commonly used in oilfields, as follows.

● Figure size: 200 × 100 pixels.
● Line width: 3.
● Line color: bule for data point 1 to 100, red for data point 101 to 200.
● X-axis range: [min**W**, max**W**].
● Y-axis range: [0, max**Z**+(max**Z**-min**Z**)*0.1].
● Ticks and tick labels: off.

**Fig. 2:**
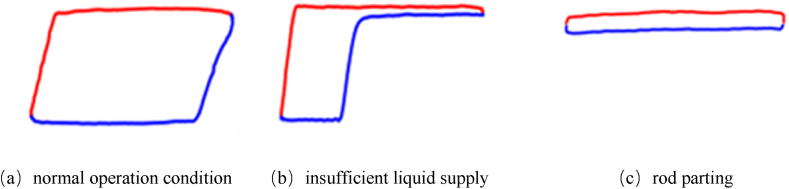
Standardized indicator diagrams.

Fig. 2 presents three examples of standardized indicator diagrams (normal operation condition, insufficient liquid supply, rod parting). The standardized drawing of indicator diagrams helps us eliminate some misjudgment of fault types. If we use default settings of drawing tool such as Python, the closed curve of rod parting will occupy the entire diagram, despite a small difference between the maximum and minimum load, which very easy confuse with normal operation condition, and should be avoided.

Images can be represented as matrices made up of pixel values. In the case of binary images, their 2-dimensional matrices consist of only two values: 0 and 1.0 represents black and 1 represents white. For grayscale images, one channel is sufficient for representation, with pixel values ranging from 0 to 255. White is represented as 255, and black is represented as 0. Therefore, black and white pictures are also called grayscale images, which are widely used in medical and image recognition fields [30]. RGB images, are 3-dimensional matrices that represent chromatic image, and require three channels to represent. Each pixel in RGB images can be represented by different colors, with each color having 0 to 255 levels of brightness. For example, blue is (0, 0, 255). The indicator diagram is an RGB image.

## 3 Feature extraction of indicator diagram

### 3.1 Feature extraction based on original vector

In this method, we directly use the displacement vector and load vector acquired by the dynamometer to form an original vector as the feature. The original vector **O** = [ w_1_, w_2_, …, w_200,_ z_1_, z_2_, …, z_200_]. Table 3 gives some examples of the processed original vector of the indicator diagram. Through concatenation, each indicator diagram corresponds to a vector with a length of 400, where 1–200 represents the displacement vector and 201–400 represents the load vector.

**Table 3:** Original vector of indicator diagram.

	1	2	3	…	199	200	201	…	388	399	400
1	0.000	0.006	0.003	…	0.012	0.012	35.99	…	35.11	35.40	35.64
2	0.000	0.006	0.003	…	0.012	0.012	36.37	…	35.34	35.60	35.93
3	6.043	6.047	6.047	…	6.021	6.043	75.15	…	75.15	74.78	74.78
4	6.043	6.047	6.047	…	6.021	6.043	74.24	…	73.66	77.78	73.78
…	…	…	…	…				…	…	…	…

The displacement vector and load vector are the raw data collected by the dynamometer, so it can be considered that this method retains all the information of the indicator diagram. Nevertheless, the feature vectors created by this method are not intuitive for human vision, and humans need to convert the feature vectors into indicator diagram before they can effectively distinguish between samples.

This method uses a combination of displacement vectors and load vectors to characterize indicator diagram information. The displacement vector and load vector are the raw data collected by the dynamometer, so it can be considered that this method retains all the information of the indicator diagram. Nevertheless, the feature vectors created by this method are not intuitive for human vision, and humans need to convert the feature vectors into indicator diagrams before they can effectively distinguish between samples.

### 3.2 Feature extraction based on three-dimensional pixel tensor

**Fig. 3:**
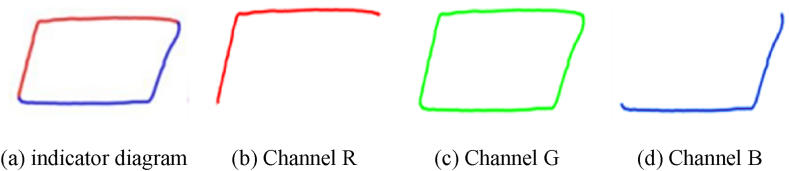
Three channels of indicator diagram.

**Fig. 4:**
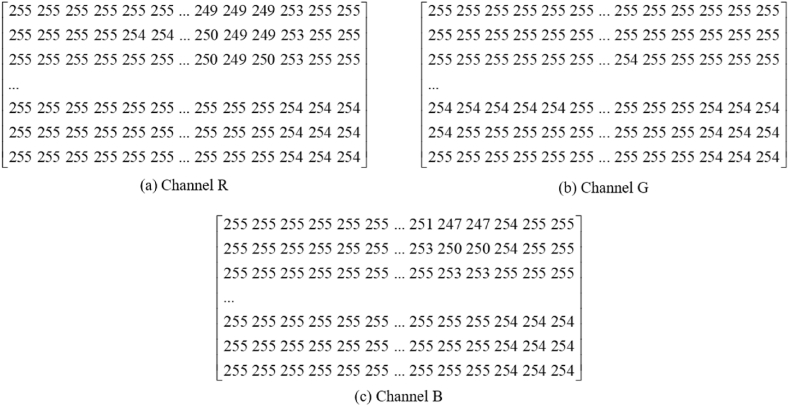
The pixel tensor of indicator diagram.

The indicator diagram is represented as a color image, which is a three-dimensional array or a tensor in a computer, with the size of 200 × 100 × 3. Among them, 3 represents three channels, corresponding to the colors red, green, and blue, respectively. A colored image consist of three colors each corresponding to a primary color channel. Take Fig. 2 (a) as example, indicator diagram can be divided by 3 channels, Fig. 3 shows the each channel. The pixel tensor is shown in Fig. 4.

Scalars are often referred to as 0-dimensional tensors, while vectors are referred to as 1-dimensional tensors. Similarly, matrices are regarded as 2-dimensional tensors, and RGB images can be represented by 3-dimensional tensors. Therefore, we can think of tensors as multidimensional arrays and the indicator diagram as three-dimensional arrays or tensors. After reading a 200 × 100 × 3 indicator diagram into a computer and converting it into a three-dimensional tensor, it contains 60000 pixel points.

In this method, the first step is to use the displacement vector and load vector to draw a standardized indicator diagram. The drawing specification follows the oilfield standard, ensuring that all the information of the indicator diagram is preserved. Unlike the feature extraction based on the original vector, this method provides more intuitive visual information. To prevent information loss, the feature vector dimension in this method reaches 60,000.

### 3.3 Feature extraction based on convolutional neural network

Convolutional neural network (CNN) is an efficient recognition method that has emerged in recent years and has garnered widespread attention and discussion in various fields [31]. A convolutional layer is composed of multiple convolution kernels, also known as the feature extraction layer. Mainly used to learn and obtain the features in the image, and the feature weight values in the convolution kernels can be automatically learned and updated. In recent years, CNN has been applied to object detection [32],medical treatment [[33], [34]], image recognition [[35], [36]], etc. We need to use CNN to extract the features of the indicator diagrams, and there are a series of publicly available pre-trained CNN models trained on the ImageNet dataset for us to choose from.

**Fig. 5:**
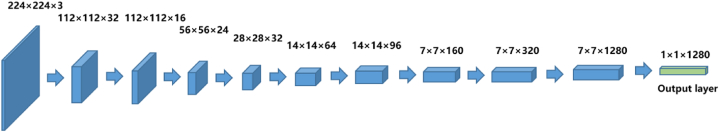
The model structure of MobileNetV2.

The ImageNet dataset is widely regarded as one of the most essential and frequently used datasets for image classification, detection and location in the field of deep learning. It comprises tens of thousands of images in diverse categories such as animals, flowers, fruits, and people. From 2010 to 2017, the ILSVRC (ImageNet Large Scale Visual Recognition Challenge) competition utilized ImageNet dataset, leading to the creation of deep learning network models like AlexNet (2012), VGG (2014), and ResNet (2015). By learning the basic features of thousands of objects on the ImageNet dataset, the CNN has formed a pre trained CNN model with weight parameters [37], which has a strong feature extraction ability. The MobileNetV2 model, as illustrated in Fig. 5, was proposed by Google in 2018 [38]. In comparison to its predecessor, MobileNetV1, the model is not only more accurate, but also more compact in size. In this study, we employ the pre-trained CNN model of MobileNetV2 to extract indicator diagram features. The model maps local features of the indicator diagram to various neurons, which are then aggregated to form global information. Finally, the output is a sequence of digital feature vectors, capable of discerning between distinct types of indicator diagrams.

The feature extraction based on CNN makes full use of the feature extraction ability of CNN. For feature extraction rather than classification, the MobileNetV2 model removes the classification layer and used to extract the characteristics of indicator diagram.

Prior to feature extraction, it is essential to normalize the indicator diagram. Normalization is a data processing technique that restricts the data between 0 and 1. Normalizing the data leads to a significant reduction in the optimal solution's fluctuations and a faster convergence rate. The normalization method can be performed as follows (assuming the *i*th column).

(1) $$\frac{x_{i}-\min\left(x_{i}\right)}{\max\left(x_{i}\right)-\min\left(x_{i}\right)}$$

where *x*_i_ is the original characteristic parameter, max (*x*_i_) represents the maximum value, and min (*x*_i_) represents the minimum value.

**Fig. 6:**
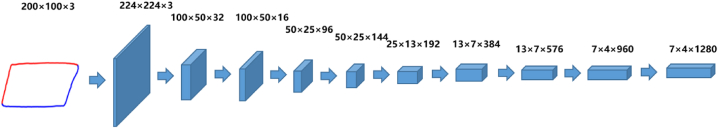
The process of feature extraction of indicator diagram.

To ensure consistency in picture size, the indicator diagram (200 × 100 × 3) undergoes preprocessing before being imported into the picture dataset, flattened and normalized to enhance the operational convergence rate. Upon inputting the image to the convolution layer, MobileNetV2 maps all the feature containing local information (including the height, width, and number of channels of the feature) to the 7 × 4 × 1280 dimensions. Fig. 6 illustrates the changes in parameters for each layer when using MobileNetV2 to extract features of indicator diagram.

The output of the model is a 7 × 4 × 1280 dimensional array, which is ultimately transformed into a vector with a length of 35840.

On the basis of method 2 (feature extraction based on three-dimensional pixel tensor), pre-trained MobileNetV2 is introduced for further feature extraction in this method. Through a sequence of operations such as neural network convolution and pooling, the information is compressed and transformed. As a result, the feature dimension is reduced from 60,000 to 35,840. However, Due to the lack of interpretability in neural networks, it is not possible to predict the level of information retention in advance. It needs to be evaluated according to the actual clustering result.

## 4 Clustering of indicator diagrams

The conversion of each indicator diagram into a vector was accomplished. Subsequently, cluster the vectors of each indicator diagram to maximize the differences between each class and minimize the differences within the class.

### 4.1 K-means clustering

Clustering mainly refers to classifying data by identifying features that can describe their correlation or differentiation [39]. The effectiveness of clustering is determined by the degree of intra-class similarity, which should be high, and inter-class dissimilarity, which should be low. In other words, the main task of clustering is to achieve high similarity within a class and low similarity between classes, making the distance between classes as large as possible, and the distance between samples within a class and class centers as small as possible. As one of the most commonly used techniques in data analysis, clustering algorithm has been widely used in pattern recognition [40], machine learning [41], image processing [42], data mining [43], etc. Algorithms for data clustering are grouped into two major categories, namely, hierarchical clustering algorithms and partitioned clustering algorithms [44]. The method based on partition being the most commonly used clustering approach. It divides all objects into mutually exclusive categories, and each object belongs to a category. Its purpose is to increase intra-cluster similarity and reduce inter-cluster similarity. Common algorithms based on partition include K-means, K-medoids, and K-prototype. In this study, the K-means algorithm was selected for feature vector clustering due to its simple calculation principle, easy implementation, high efficiency, and success in various fields. K-means algorithm is currently the most widely used method in clustering.

K-means clustering counts the distance between the data object and the cluster centroid, and data objects close to the centroid are divided into the same category.

The specific steps of K-means algorithm are.

(1) Specify K initial centroids, each of which is a class.
(2) For each remaining sample, count their distances from all centroids, and then put them into the nearest cluster.
(3) After the division, recalculate the centroids of each class, and then count the distance from each sample to the centroids of each class to reclassify each sample.
(4) Repeat steps (2) and (3) until the centroid does not change.

However, different cluster centers may produce different clustering results. We must overcome the negative impact of the selection of initial cluster centers on the clustering results, make the initial cluster centers as far away from each other as possible, and use K-means++ algorithm to optimize.

The specific steps of K-means++ algorithm are.

(1) A sample is randomly selected as the first initial cluster center in the sample set.
(2) Calculate the distance *D*(*x*) from other samples *x* to the nearest cluster center.
(3) Calculate the probability *P*(*x*) that all samples become the next cluster center, as follows:

(2) $$P\left(x\right)=\frac{D{\left(x\right)}^{2}}{\sum D{\left(x\right)}^{2}}$$

(4) Select the sample corresponding to the maximum probability value as the next cluster center.

Repeat steps (2), (3) and (4) until K cluster centers are selected.

### 4.2 Distance measurement

Common distance measures include Euclidean distance, Manhattan distance, Chebyshev distance [45]. The K-means algorithm in the deep learning library sklearn encapsulates Euclidean distance for distance measurement, because Euclidean distance can be used as the distance calculation problem of any spatial structure, representing the actual length between two points in an n-dimensional spatial structure, or the actual length of a vector to the origin, while the Euclidean distance in a 2-dimensional or 3-dimensional space refers to the actual distance between two points. The Euclidean distance *d* of points *x* and *y* is：

(3) $$d\left(x,y\right)=\sqrt{\sum_{i=1}^{n}{\left(x_{i}-y_{i}\right)}^{2}}$$

where *n* is the number of sample points contained by *x* or *y*, *x*_i_ and *y*_i_ represent the *i*th sample point.

### 4.3 Evaluation index of clustering

The K-means algorithm requires optimization to ensure that samples within the same category exhibit high similarity [46]. There are currently many indicators for evaluating clustering effectiveness, with the most commonly used being SSE, SC, and H.

SSE (sum of squares due to error):

(4) $$SSE=\sum_{i=1}^{K}\sum_{x\in K_{i}}{\left(c_{i}-x\right)}^{2}$$

where *K* is the number of clusters, *x* is the point in *K*_i_, *K*_i_ is the *i*th category, and *c*_i_ is the centroid of the *i*th category.

Silhouette Coefficient (SC) is one of the main indexes to evaluate the clustering results [47], which is divided into cohesion and separation, and can be understood as describing the definition of the contour of each category after clustering. Cohesion can be understood as reflecting the similarity between samples and elements within the class, while separation can be understood as reflecting the differences between samples and elements outside the class. The equation of SC is as follows:

(5) $$SC=\frac{b\left(i\right)-a\left(i\right)}{\max\left\{a\left(i\right),b\left(i\right)\right\}}$$

where *a*(*i*) represents the cohesion of the sample, that is, the average of the compactness of vector *I* to other vectors in the same cluster, which is calculated as follows:

(6) $$a\left(i\right)=\frac{1}{n-1}\sum_{j\neq i}^{n}d\left(i,j\right)$$

where *n* represents the number of clusters, *j* represents other samples in the same class as sample *i*, and distance represents the distance between *i* and *j*. The smaller *a*(*i*) is, the closer the class is. *b*(*i*) represents the dissimilarity between clusters, that is, the average dissimilarity between vector *i* and other clusters, which is calculated as follows:

(7) $$b\left(i\right)=\min\left[\frac{1}{n-1}\sum_{j\neq i}^{n}d\left(i,j\right)\right]$$

The *S*(*i*) is between [−1,1]. The larger the SC, the better the clustering effect.

If a cluster contains only one category of indicator diagrams, it satisfies Homogeneity (*H*). In fact, it can also be considered as accuracy (the proportion of correctly classified samples in each cluster to the total number of samples in that cluster). The equation of *H* is as follows:

(8) $$H=\frac{1}{K}\sum_{i=1}^{K}\frac{N\left(R_{i}=K_{i}\right)}{N\left(K_{i}\right)}$$

where *K* is the number of clusters, *K*_i_ is the *i*th category, and *R*_i_ is the correctly classified samples of the *i*th category. *N* represents the number of samples.

### 4.4 Pseudo codes of the proposed methods

Combining the three feature extraction methods with the K-means clustering algorithm introduced in this section, three clustering algorithms using different feature extraction strategies are obtained. The pseudo codes of the algorithms are shown in Algorithm 1 to 3.

**Figure:**
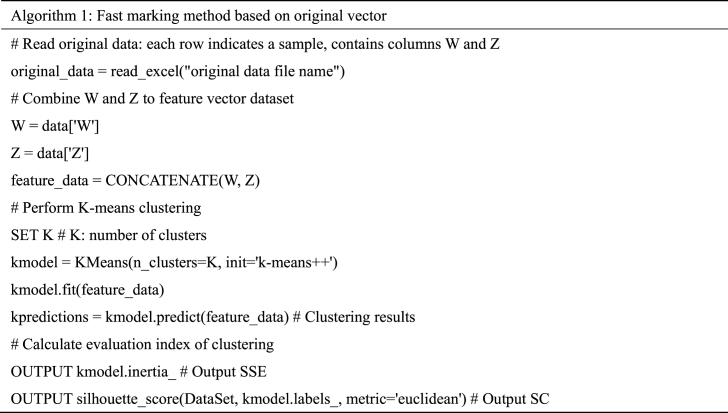
Pseudo code of the algorithm 1.

**Figure:**
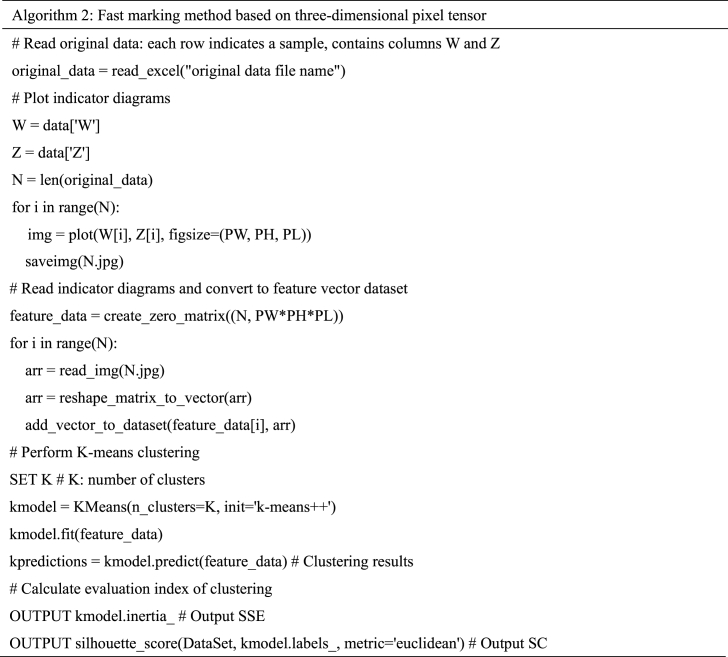
Pseudo code of the algorithm 2.

**Figure:**
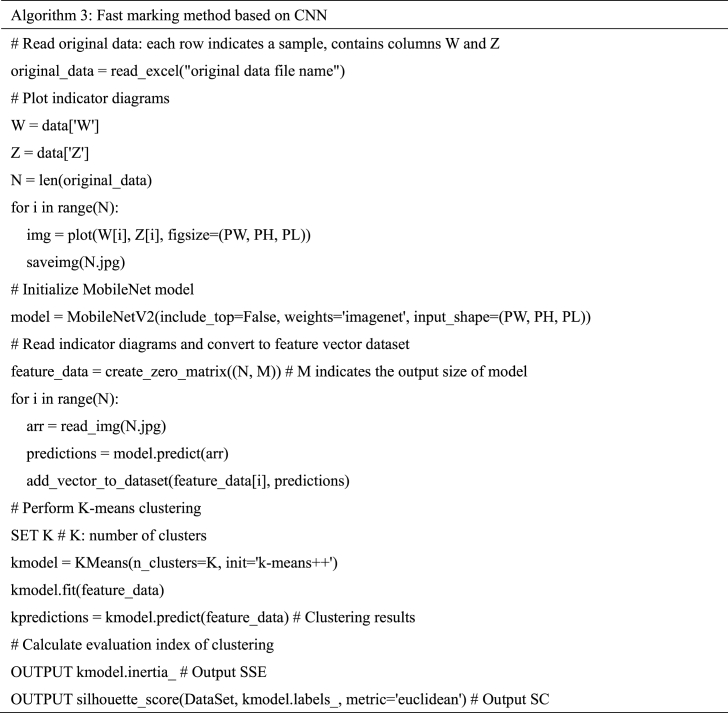
Pseudo code of the algorithm 3.

## 5 Experimental results and analysis

### 5.1 Experimental environment

Hardware environment: Intel (R) Core (TM) i7-6700HQ CPU @ 2.60 GHz, memory 8G 1 T hard disk. Software environment: Window10 64 bit operating system, PyCharm Community Edition 2021.2.

### 5.2 Experimental data

From a pool of 2000 pumping wells, 100 wells were randomly selected and 200 samples were collected from each, resulting in a total of 20000 samples. Each sample contained one displacement vector with a length of 200 and one load vector with a length of 200. The dimension of drawn indicator diagrams were 200 × 100 × 3.

Three feature extraction methods were applied, resulting in 20000 feature vectors for each method. Method 1 produced feature vectors with a length of 400, Method 2 produced feature vectors with a length of 60000, and Method 3 produced feature vectors with a length of 35840.

### 5.3 Experimental result

For the 20000 samples mentioned above, the K-means algorithm is employed for clustering, where the number of clustering categories is set to 80, corresponding to 80 folders. Upon completion of the clustering process, the indicator diagrams, numbered 0–19999, are placed in the respective folders based on their clustering results.

Clustering the original vector data in Table 3, the category will be saved in the last column, as shown in Table 4. Next, the vector will be drawn into standardized indicator diagram and saved in appropriate folders, partial indicator diagrams of six categories illustrated in Fig. 10.

**Table 4:** Vector clustering results.

	1	2	3	…	398	399	400	category
0	0.0	0.006	0.006	…	26.39	26.77	27.56	0
1	0.0	0.006	0.006	…	27.20	27.76	28.61	0
2	0.0	0.010	0.013	…	75.72	75.81	75.84	1
3	0.0	0.010	0.013	…	75.48	75.48	75.57	1
…	…	…	…	…	…	…	…	…

Table 5 presents the comparison of the time consumption of the three different indicator diagram clustering methods discussed in this paper. The total time includes the time consuming of feature extraction and clustering.

**Table 5:** Comparison of time consuming.

Feature extraction methods	Time consumption for feature extraction/(h)	Clustering time/(h)	Total time consumption/(h)
original vector	0.052	0.183	0.235
three-dimensional pixel tensor	0.103	8.163	8.266
CNN	0.267	0.385	0.652

**Fig. 7:**
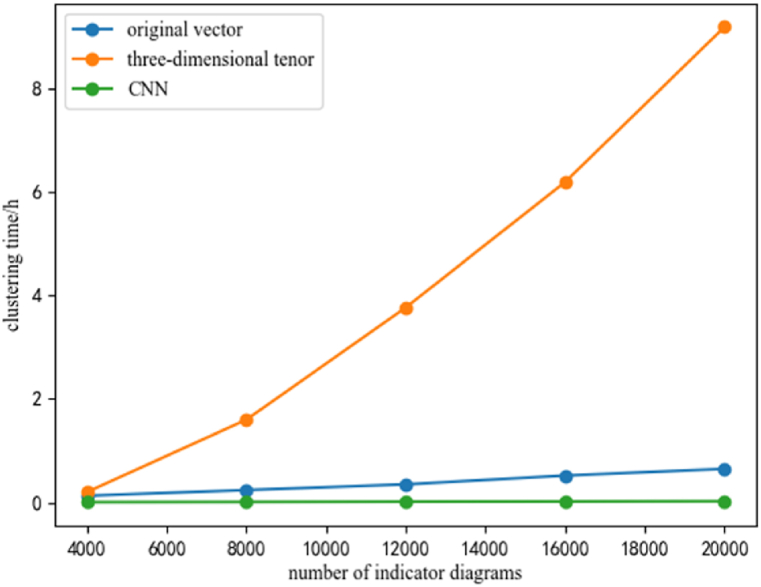
Relationship between the number of indicator diagrams and clustering time.

In order to investigate the impact of different sample numbers on clustering time, a range of 4000–20000 samples was examined, and the clustering time was calculated for each. Fig. 7 illustrates the changes in clustering time as the number of samples increases.

**Fig. 8:**
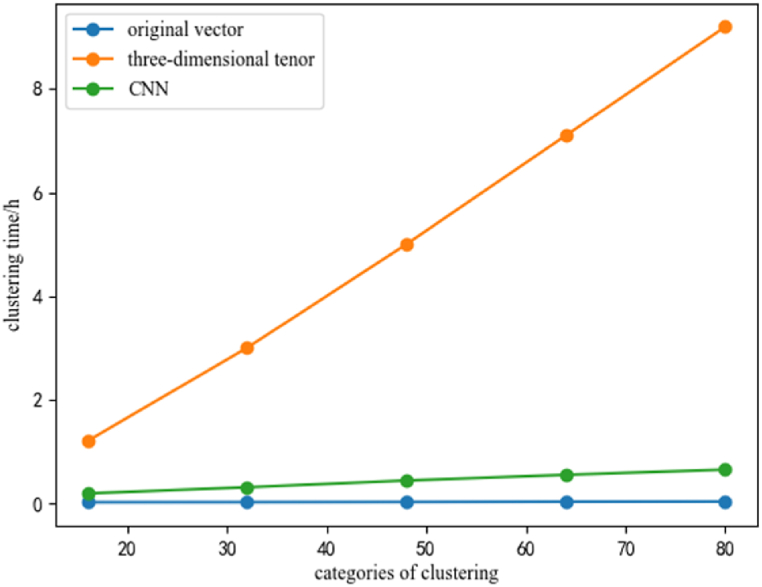
Relationship between the categories of clustering and clustering time.

In order to analyze the impact of different clustering categories on the clustering time, a range of 16–80 categories was examined, and the clustering time was calculated for each. Fig. 8 illustrates the changes in clustering time as the number of categories increases.

As depicted in Fig. 7, Fig. 8 that with the increase of the number of indicator diagrams or the increase of the clustering categories, the clustering time of feature extraction based on three-dimensional pixel tensor increases rapidly and takes a long time, which does not meet the research purpose of fast clustering for indicator diagrams. Additionally, it can be seen from Fig. 7, Fig. 8 that these two periods are exponential growth and linear growth.

**Fig. 9:**
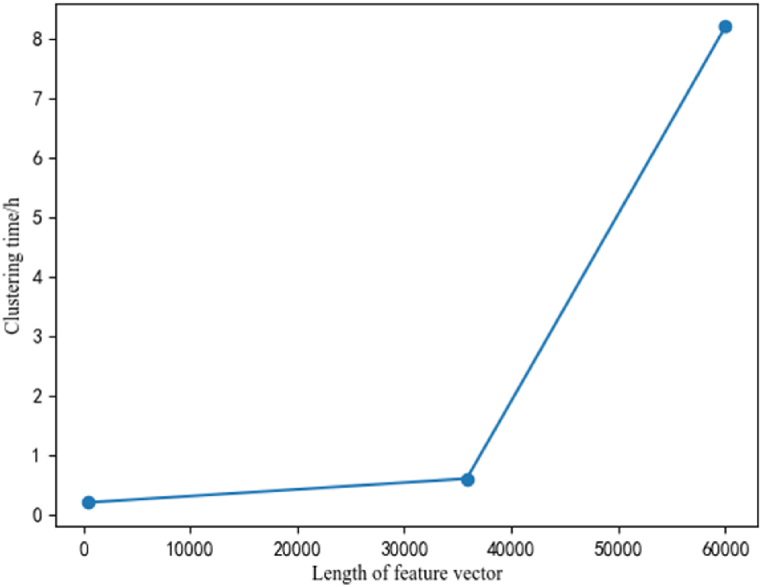
Relationship between feature vector length and clustering time.

Considering that the feature vector lengths obtained by three feature extraction methods are different, the relationship between feature vector length and clustering time is plotted as shown in Fig. 9. From the figure, it can be seen that as the feature vector length increases, the clustering time also increases, and the increase rate follows an exponential relationship. This further indicates the importance of selecting an appropriate feature extraction method to improve clustering efficiency.

**Fig. 10:**
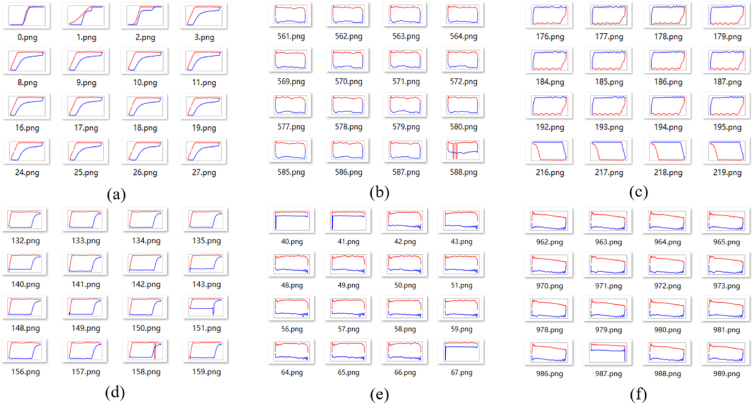
Partial clustering results of original vector.

**Fig. 11:**
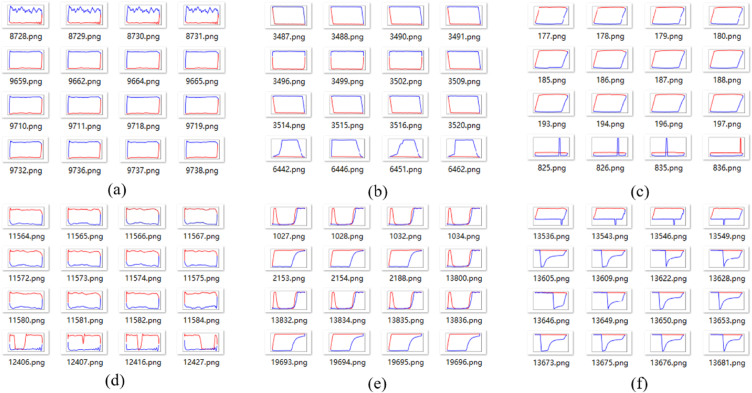
Partial clustering results of three-dimensional tensor.

**Fig. 12:**
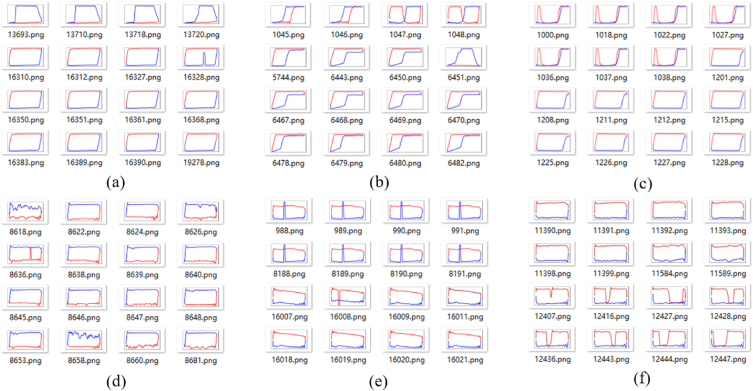
Partial clustering results of pre-trained CNN.

It is worth noting that clustering of indicator diagrams may lead to certain errors, whereby indicator diagrams of different types are sometimes grouped together in the same folder. Nonetheless, the majority of folders achieve correct clustering. Six folders with incorrect clustering were selected for each of the three methods, as demonstrated in Fig. 10, Fig. 11, Fig. 12.

From an intuitive perspective, among the folders with clustering errors, the first method has the least proportion of samples with errors. To further measure the clustering, we employ the *H* as the average accuracy for each method. After statistical analysis of each folder, the distribution of accuracy of the three methods are shown in Fig. 14. After calculation, the *H* of the three methods are 98%, 92%, and 95%, respectively.

As can be seen from Fig. 14, the first clustering method has the largest number of folders with an accuracy of 100%, and the lowest number of folders with an accuracy rate of less than 80%. Additionally, the accuracy of the first method is predominantly distributed above 90%, with a comparatively low number of folders displaying an accuracy rate below 90%. These results indicate that the first clustering method is a highly reliable and accurate approach, producing a considerable number of precise outcomes with minimal discrepancies. Fig. 7, Fig. 8 shows that the Feature extraction based on three-dimensional tensor takes significantly longer compared to the other two clustering methods. Furthermore, Fig. 14 reveals that the second method does not exhibit the highest number of folders with an accuracy of 100%, while has a higher number of folders with an accuracy rate of less than 80%.

In addition, the *SC* for the three methods are 0.53, 0.20, and 0.23, respectively. This indicates that the higher the *H*, the larger the *SC*. From the *SC*, feature extraction methods based on original vectors also demonstrated its advantages.

**Fig. 13:**
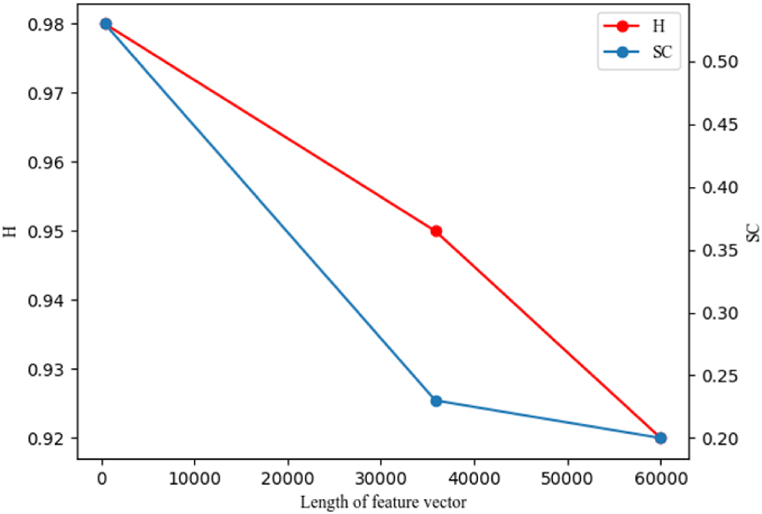
Relationship between length of feature vector and clustering accuracy.

Considering that the feature vectors obtained from three different feature extraction methods have different lengths, the relationship between the length of the feature vectors and the clustering accuracy metrics H and SC is plotted in Fig. 13. From the figure, it can be observed that method 1, with a feature vector length of only 400, achieves the highest H and SC values, indicating the best clustering accuracy. Method 3, with a feature vector length of 35,840, performs second best, followed by method 2 with a feature vector length of 60,000. This suggests that the clustering accuracy is not directly related to the length of the feature vectors, but rather to the amount of information preserved within them.

**Fig. 14:**
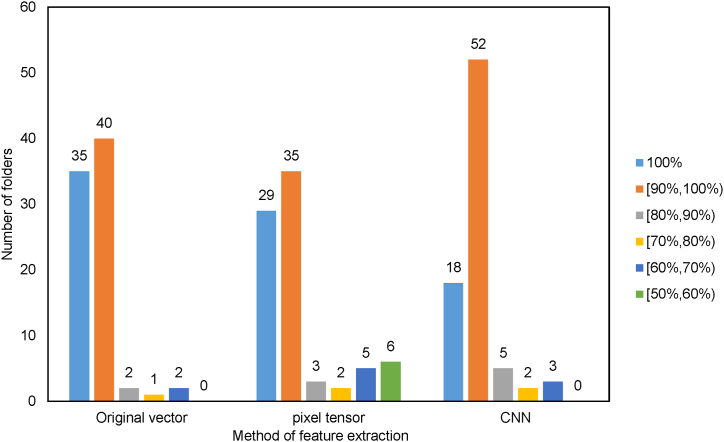
Distribution of accuracy.

Although method 2 has the longest feature vector length, over 90% of the vector consists of zeros, resulting in low information density and lower clustering accuracy. Method 3 compresses the feature vectors based on method 2, sacrificing some information but increasing information density, thereby improving clustering accuracy compared to method 2. Furthermore, both method 2 and method 3 initially transform the original data into indicator diagrams for visual information extraction. However, from the perspective of clustering results, although visual approaches are more intuitive and easier for humans to interpret, these approaches does not show superiority for clustering algorithm.

Due to the extensive computation and long time consuming, the second method does not exhibit better clustering results than other methods. Therefore, further research on this method is not considered. Different feature extraction methods can be compared from a macro perspective by clustering different numbers of samples into the same categories or clustering the same number of samples into different categories. The findings of these two experimental schemes are summarized in Table 6 and Table 7.

**Table 6:** Comparison of clustering effects of different sample quantities.

Feature extraction based on CNN						Feature extraction based on original vector				
Quantities	4000	8000	12000	16000	20000	4000	8000	12000	16000	20000
Categories	80	80	80	80	80	80	80	80	80	80
SSE, × 10^7^	1.83	4.51	7.75	11.40	14.50	0.102	0.253	0.752	1.48	2.05
SC	0.21	0.27	0.25	0.24	0.23	0.55	0.68	0.66	0.59	0.53

**Table 7:** Comparison of clustering effects of different categories.

Feature extraction based on CNN	Quantities	20000	20000	20000	20000	20000	20000	20000	20000	20000	20000
	Categories	16	32	48	64	80	96	112	128	144	160
	SSE, ×10^8^	2.47	2.05	1.7	1.59	1.45	1.35	1.27	1.21	1.18	1.15
	SC	0.11	0.14	0.18	0.20	0.23	0.24	0.25	0.25	0.24	0.24
**Feature extraction based on original vector**	Quantities	20000	20000	20000	20000	20000	20000	20000	20000	20000	20000
	Categories	16	32	48	64	80	96	112	128	144	160
	SSE, ×10^8^	0.998	0.535	0.355	0.262	0.205	0.164	0.136	0.115	0.099	0.087
	SC	0.36	0.40	0.46	0.50	0.53	0.57	0.59	0.61	0.63	0.63

Table 6 shows that with the increase of sample quantities, the clustering accuracy is decreasing. Table 7 shows that with the increase of the number of categories, the clustering accuracy is increasing. At this point, *SSE* continues to decrease, and *SC* reaches its maximum value, indicating that *SC* can be used to assist in determining the optimal clustering category. From Tables 7 and it can be seen that the best clustering category obtained by the first method is 112, the best clustering category obtained by the second method is 144, and the second method has a more detailed division of the dataset.

**Fig. 15:**
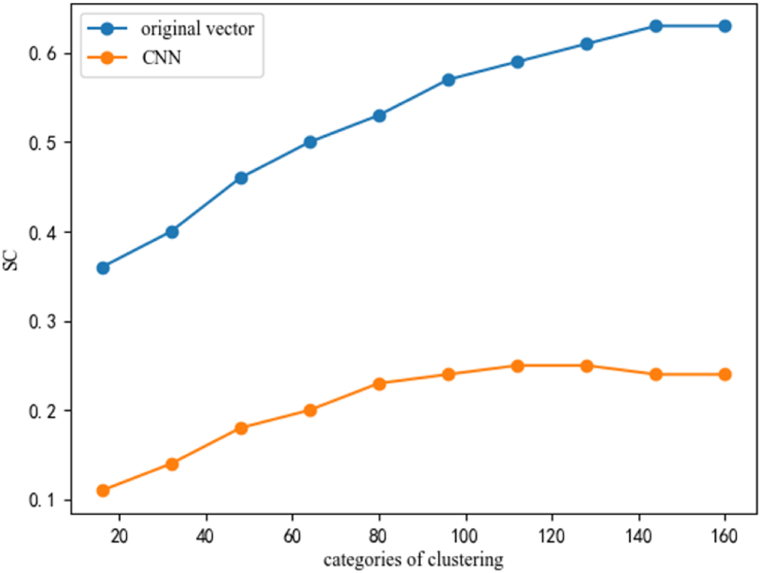
Changes in two methods of SC.

Fig. 15 shows the changes in *SC*. As the number of categories increases, we observed that the *SC* initially increases with the number of categories. However, beyond a certain threshold, the *SC* reaches a plateau and no longer improves. Based on the comparative analysis of the two clustering methods presented in Table 6, Table 7, it can be concluded that the clustering method based on the original vector yields a smaller *SSE*, and a larger *SC*. These findings suggest that the clustering effect of this method is better.

The effect of this study was further analyzed based on practical application. If 20,000 well samples need to be marked for working condition type, it takes an experienced oilfield engineer approximately 20 seconds to mark one sample, resulting in a total time of approximately 111.111 h to complete the marking process. However, if the fast marking method based on original vector proposed in this study is used to assist in the marking, previous tests have shown that the clustering of 20,000 samples can be completed in just 0.235 h with an accuracy of 98%. Only 400 samples would require manual intervention, which would take approximately 2.222 h. Consequently, the entire process can be completed in just 2.457 h. Compared with manual labeling, the proposed method in this study can achieve a 45-fold increase in efficiency.

## 6 Conclusions

The traditional sample set preparation for fault diagnosis of pumping wells relies on expert experience for marking, which is both time-consuming and labor-intensive. This research proposed three fast marking methods, each of which adopts a different feature extraction strategy and is coupled with K-means clustering algorithm.

The performance comparison analysis of the methods was conducted through experimental testing. The total time consumed by the three methods was 0.235h, 8.266h, and 0.652h, respectively. The accuracy measures for the three methods, H and SC, were 98%, 92%, 95% and 0.53, 0.20, 0.23, respectively. Method 1 is the most outstanding in terms of efficiency and accuracy among the three methods.

For indicator diagram clustering task, the clustering time consumption is influenced by the number of samples, cluster categories, and feature dimension. It increases with an exponential speed as the number of samples and feature dimensions increase, while it has a linear relationship with the number of cluster categories. However, there is no direct correlation between clustering accuracy and feature dimension. Clustering accuracy depends more on the amount and density of information. Although plotting the original data into indicator diagram is more visually distinguishable by humans, this method does not achieve better accuracy for clustering algorithm.

According to practice, the efficiency of pumping well working condition marking by using the clustering algorithm proposed in this study can be increased by 45 times compared with manual marking. Nevertheless, clustering too many samples at once may still result in excessive computational load and prolonged calculation time. In addition, there is still a lack of clear criteria for determining the number of clustering categories.

The proposed method can also be applied to other sample marking problems, such as well logging interpretation. It is important to note that data or images characterize for other problems may differ from the indicator diagram, which could result in biased accuracy and efficiency.

Future research can focus on improving algorithmic efficiency, exploring fast marking for large sample sets, addressing sample imbalance, and balancing computational efficiency with information preservation.

## Data availability statement

Data will be made available on request.

## CRediT authorship contribution statement

**Xiang Wang:** Validation, Supervision, Project administration, Funding acquisition, Data curation. **Zhiwei Shao:** Writing – review & editing, Writing – original draft, Visualization, Investigation. **Yancen Shen:** Writing – review & editing. **Yanfeng He:** Writing – review & editing.

## Declaration of competing interest

The authors declare that they have no known competing financial interests or personal relationships that could have appeared to influence the work reported in this paper.
